# Multiple myeloma with unexplained isolated anaemia in a 24year old man- a case report

**DOI:** 10.4314/ahs.v22i4.9

**Published:** 2022-12

**Authors:** Oladapo Wale Aworanti, Sunday Peter Ogundeji, Olateni Asake Adeoye, Wuraola Adebola Shokunbi

**Affiliations:** 1 University College Hospital Ibadan, Department of Haematology; 2 University of Ibadan College of Medicine, Department of Haematology

**Keywords:** Multiple myeloma, isolated anaemia, young patient

## Abstract

**Background:**

Multiple myeloma (MM) is a disease of the elderly with a median age at presentation of 70 years. It is rare to diagnose MM in individuals less than 40 years and even extremely rare in those less than 30 years of age. MM is usually suspected in those aged 50 years and above having a combination of hypercalcemia, renal insufficiency, anaemia and bone lesions. Although anaemia is a common clinical feature of MM, it is very rare that anaemia would be the only clinical presentation, hence the need to report this index case.

**Case Presentation:**

We present a rare case of MM in a 24-year- old male who presented with only symptomatic anaemia. Investigations for the cause of anaemia, including Bone marrow aspiration cytology revealed a diagnosis of MM ISS stage II. Here, we highlighted the need to seek early haematologist consultation in investigating patients' whose cause of anaemia is not immediately obvious from the clinical presentation and routine laboratory investigations.

**Conclusion:**

MM can present at a younger age with unexplained anaemia without bone pains or renal insufficiency. High level of suspicion for MM is required in young patients with unexplained anaemia

## Introduction

Multiple Myeloma (MM) is a B cell malignancy characterized by monoclonal expression and accumulation of abnormal plasma cells in the bone marrow^1^,^2^. Multiple myeloma belongs to a group of blood disorder referred to as Plasma cell dyscrasia, other disease components in the group include Benign Monoclonal disease such as Monoclonal Gammopathy of Undetermined Significant (MGUS), Indolent Lymphoma and Heavy chain diseases. In Caucasians, MM constitutes about 1% of all malignancies, and it is 2^nd^ most common blood cancer, accounting for about 10% of haematological malignancies^3^. In Nigeria, MM accounts for about 9% of all hematological malignancies and it is ranks 4th after chronic myeloid leukemia, non-Hodgkin's lymphoma, and chronic lymphocytic leukemia in frequency.^1^ ^4^,^5^

The male: female ratio ranged between 1.1: 1 to 4:1^1^,^3^,^4^ and it is commoner in blacks 6. The incidence of Multiple myeloma increases with increasing age worldwide, with a median age at presentation of 60 years. This is due to a reduction in the ability of the elderly immune system to clear potential myeloma precursors. In MM, there is clonal proliferation of plasma cells, these cells are confined to the bone marrow but they may also be seen in the peripheral blood in end stage myeloma or in Plasma cell leukaemia.^3^

Myeloma cells are long lived plasma cells which have been exposed to antigen stimulation, having undergone B cell maturation processes. They are post germinal centre plasma cells which have undergone immunoglobulin gene recombination, class switching and somatic hyper- mutation, and subsequently home to the marrow.

The causative factor of MM is unknown but exposure to chemicals like dioxins, solvents, radiation and viral infections (Hepatitis, HIV, EBV, Herpes and Cytomegalovirus) have been implicated in the pathogenesis^6^.

MM patients present with different complaints but the dominating symptoms consist of bone pains, features of anaemia and kidney disease^2^,^3^,^4^. About 96% of MM patients in our institution had low back and waist pain.^3^

The purpose of this report is to highlight the unusually young age of the patient, who presented with a four months history of isolated anaemia.

## Case report

He is a 24-year- old man who presented with about 4 months history of progressive exercise intolerance and breathlessness on mild exertion. There was an episode of fainting spells. No history suggestive of acute or chronic blood loss or haemolysis. There was also no bone pain, facial swelling and no reduction in urinary output or other features suggestive of renal impairment. No history of cardiac abnormalities.

At the onset of the illness he was managed in a private facility for an infection (typhoid enteritis). He was transfused with two units of blood at a packed cell volume (PCV) of 12%.

He is not a known hypertensive, diabetic, asthmatic or PUD patient. His haemoglobin phenotype is A. He is not on any routine medications but takes herbal concoction. He does not smoke cigarette nor take alcoholic drinks. He works as a fashion designer though he had Ordinary National Diploma in Civil Engineering.

Examination at presentation showed a young man in no obvious distress, severely pale, anicteric, afebrile (T-37°C), a cyanosed, fair hydration status with no significant peripheral lymphadenopathy.

Cardiovascular system examination showed a pulse rate of 112 beats per minute, blood pressure of 110/60mmHg, 1st, 2nd and 3rd heart sound were heard with gallop rhythm.

Physical examination of the Chest, Abdominal, and Central Nervous system were normal.

Laboratory investigations include Complete Blood count: Haematocrit- 15%, White Blood Cell count- 6,880cells/mm3, platelets of 113,000cells /mm3. White cell differential was as follows: Neutrophil: 46%, Lymphocyte: 40%, Monocyte: 13%, Eosinophil: 1%. The haematological investigations are as shown in Table I.

**Table I:** Haematological parameters of the patient

Parameters	At Presentation	Week one	Week two
Haematocrit (%)	15.0	*21.0	18.0
White blood cell count (c/mm^3^)	6,880	7,700	7,900
Absolute Neutrophil count (c/mm^3^)	3,160	3,800	3,700
Absolute Lymphocyte count (c/mm^3^)	2,710	2,500	3,000
Absolute Monocyte count(c/mm^3^)	920	1,000	980
Platelet count(c/mm^3^)	113,000	127,000	154,000
ESR (mm in 1^st^ hour)	>130	-	-
Reticulocyte count (%)	3.1	-	-
Absolute Reticulocyte count (x10^9^/L)	73.47	-	-
Corrected reticulocyte count (%)	1.5	-	-
Direct coombs Test	Negative	-	-

* Post two units of red cell concentrate

The review of peripheral blood film showed normocytic normochromic red cells with marked rouleaux. The white cell morphology was normal and platelets were adequate. He was urgently transfused with two units of group identical Blood group- B Rh “D” positive and compatible packed cells.

Bone Marrow Aspiration cytology showed abnormal plasmacytosis constituting about 80% of the nucleated marrow elements, and the plasma cells were of varying morphology and stages of differentiation. There was nuclear-cytoplasmic dissociation of the plasma cells.

Bone marrow histology showed hypercellularity with fat: cell ratio of 1%: 99%. The bone marrow cellular elements were nearly completely replaced by pleomorphic plasmacytoid cells.

Other investigations done include Electrolytes, Urea, Creatinine, Liver function test, as shown in Tables II, III and IV.

**Table II:** Electrolytes, Urea, Creatinine and Liver Function Test Profile of the Patient

Parameters	Value	Reference range
Sodium	137mmol/L	135–145
Potassium	3.3mmol/L	3.5–5.0
Bicarbonate	21mmol/L	20–30
Chloride	101mmol/L	95–110
Urea	11mg/dl	15–45
Creatinine	0.9mg/dl	0.5–1.5
Calcium	11.2 mg/dl	8.5–10.0
Phosphate	4.0 mg/dl	2.5–4.5
Uric acid	7.2mg/dl	2.0–7.0
Total Protein	13.2g/dl	6.0–8.0
Albumin	2.5g/dl	3.5–5.0
Alanine Transaminase	27IU/L	0–40
Aspartate Transaminase	42 IU/L	0–37
Alkaline Phosphatase	53 IU/L	40–130
GGT	35 IU/L	7–50
Total Bilirubin	0.5mg/dl	0.2–1.0
Direct Bilirubin	0.4mg/dl	0.0–0.4
β2 microglobulin	4.8mg/dl	<2.4

**Table III:** Serum Protein Electrophoresis

Parameters	Value	Normal reference
S-Alpha 1 Globulin	4g/L	2–6
S-Alpha 2 Globulin	7g/L	3–10
S-Beta 1 Globulin	4g/L	3–6
S-Beta 2 Globulin	2g/L	2–6
S-Gamma Globulin	2g/L	6–15
S- ‘M’ Component	89g/L	0

SPE showed a monoclonal peak in the late gamma region measuring approximately 89g/L

**Table IV:** Immunoglobulin Quantitation Profile of the Patient

Parameters	Results	Reference range
S-IgA	0.26g/L	0.41–3.49
**S-IgG**	**112.30g/L**	**6.5–16.0**
S-IgM	0.40g/L	0.50–3.00

Immunoglobulin qu antitation showed immune paresis with markedly elevated IgG fraction of 112.3g/L (Ref: 6.5- 16g/L)

A diagnosis of IgG myeloma, ISS stage II was made. The patient was duly counselled about the diagnosis, disease spectrum/course, and treatment options were discussed including autologous haematopoietic stem cell transplantation. The choice of induction chemotherapy was Velcade, Thalidomide and Dexamethasome (VTD) as per standard protocol.

## Discussion

Multiple myeloma is a common haematological malignancy which affects predominantly the elderly, mainly as a result of the inability of the immune system of the elderly to clear the myeloma precursors 3. Previous reports from Nigeria showed that the age range at presentation is 54 to 62 years and that there is increased incidence with increasing age as reported globally. Previous studies reported that only 15% of MM patients presented at an age less than 55years and very few cases presented at age less than 40years 3,4. In this report, we highlighted the presentation of a 24year-old man diagnosed with MM in our facility, an unusual presentation because only less than 2% of cases are diagnosed below 40years. Muhammad et al reported a 20-year-old female diagnosed with MM who presented with a bleeding disorder due to acquired factor X inhibitor.^7^

The index patient presented only with features of recurrent anaemia about four months prior to presentation despite the extensive bone marrow involvement. Pathogenesis of anaemia in MM is related to bone marrow suppression of normal haemopoiesis and renal impairment leading to reduced erythropoietin secretion. Anaemia is a component of the diagnostic criteria for MM (CRAB) 6. There are however no other features suggestive of bone disease, renal impairment or hypercalcemia in this patient. Isolated anaemia has been previously reported in MM but this has to be supported by rouleaux formation of the red cells in the peripheral blood film review, when this is combined with isolated anaemia, there should be high index of suspicion for MM 3. Previous studies done showed that most patients with MM presents with bone pain 1–4,8–9.

Bone marrow cytology in this patient showed that 80% of nucleated marrow elements were plasma cells. Bone marrow histology showed that the marrow is completely replaced by plasma cells. Bone marrow plasmacytosis of only 10% and above with other two major criteria is diagnostic of MM 3,8,9 This underscores the importance of early bone marrow studies in patients with unexplained anaemia as this will enable the physician in streamlining the investigations.

Serum protein electrophoresis and Immunoglobulin quantitation showed that there was a monoclonal peak at late gamma region measuring 89g/L and increased IgG with immune paresis. In a previous study done in our facility, IgG MM has been found to be the commonest type and this is also consistent with other previous studies on MM 3,6

The patient also had elevated β2 microglobulin of 4.8mg/dl, a level that puts the patient in ISS stage II despite the absence of other features suggestive of Bone and/or renal disease 6

## Conclusion

Multiple myeloma can present at a younger age with unexplained isolated anaemia without features of involvement of bone or kidney. High level of suspicion for MM is required in young patients with isolated anaemia.

## Figures and Tables

**Figures Ia and Ib:**
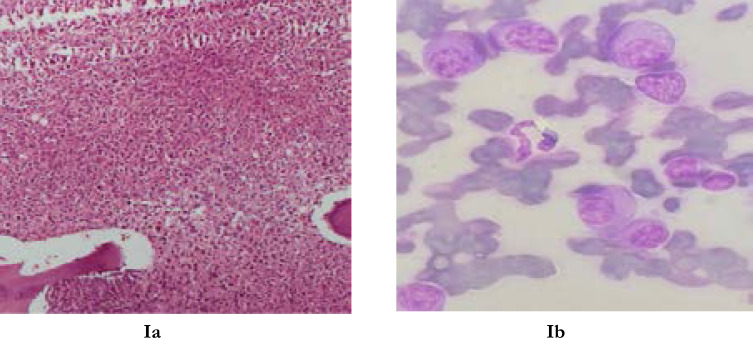
Show bone marrow cytology and histology respectively

## Data Availability

Not applicable

## Limitations

No genetic studies were done for the patient, as our health care centre do not have the necessary equipment to do this investigation and the patient is not financially buoyant to send the sample outside the country.
